# Report of a Case of Labor with Double Uterus and Vagina

**Published:** 1903-02

**Authors:** Henry T. Williams

**Affiliations:** Rochester, N. Y., Surgeon to Rochester City Hospital and St. Mary’s Hospital


### CLINICAL REPORT.

### Report of a Case of Labor with Double Uterus and Vagina.

By HENRY T. WILLIAMS, M. D., Rochester, N. Y.,
Surgeon to Rochester City Hospital and St. Mary's Hospital.

THIS patient, 26 years of age, consulted me first in June, 1896. She then had been married about eighteen months, and came to see me on account of a troublesome leucorrhea with smarting pain in urinating. Examination revealed some inflamed urethral caruncles and two distinct vaginæ, the left smaller than the right, and two distinct cervices uteri, into each of which a sound could be passed 2½ inches. The sounds could not be made to touch each other while in the uteri, showing two distinct cavities, practically two uteri, but only one ovary and tube could be felt on each side. Each vagina had a distinct hymen, the wall between the two vaginæ being about ¼ of an inch thick and extending up between the two cervices. No communication, whatever, existed between the two vaginæ. The cervices projected about ¾ of an inch into each vagina.

I advised operation for the removal of the vaginal septum, but this she would not consent to have done. She consulted me again in the latter part of December, 1896, saying she had missed her period and feared she was pregnant. I examined her in January and found both cervices slightly softened and bluish in color and the uteri somewhat enlarged. She complained of morning sickness and other symptoms of pregnancy. I could not tell if she were pregnant in one or both cavities of uterus. I again urged upon her the necessity of an operation, fearing that if she went on to delivery with such a septum, it might result in a rent into the bladder, rectum or pelvic tissue.

She consented to the operation, and on January 30, I dissected out the partition between the two vaginæ, beginning at its upper portion. I removed in one piece the entire septum from between the cervices and out to the perineum, sewing the denuded surfaces together with one continuous suture of catgut, beginning about an inch below the meatus and ending at perineum, thus making one vagina. I then introduced a large-sized hard rubber vaginal dilator, which was left in for five days, the urine being drawn through a catheter every six hours during this time; the dilator was then removed and patient allowed to urinate naturally. The parts healed by first intention and pregnancy continued normally.

Labor began on the morning of October 3, 1902, and when I reached the house, about 1 o'clock in the afternoon, labor was well advanced, and I could feel but one os uteri which was three-quarters dilated. The labor was perfectly normal and was completed in one and a half hours after I reached the house. The child was a male and weighed 7½ pounds. There was but one placenta and that came away a few minutes after the birth of the child. She made an uneventful recovery. I examined her two months afterward and found but one cervix, which was somewhat larger than an ordinary cervix would be, and one os also quite large, and the sound showed that there was but one cavity to the uterus, the partition having been completely obliterated during the labor. The vagina and perineum were intact. She has been well since that time.

---

THE SALT PACK IN RHEUMATOID ARTHRITIS.— Jonathan Hutchinson, F. R. S. (*Polyclinic*, January, p. 45) knows no remedy so almost universally effectual in removing irritation and synovial effusion in rheumatoid arthritis as the salt pack. It consists of flannel soaked in a saturated solution of common salt, which is wrapped round the affected joint, covered with oiled silk and a bandage, and kept on the whole night. It should be applied every night until the cure is effected.

